# β-arrestin2 in Infiltrated Macrophages Inhibits Excessive Inflammation after Myocardial Infarction

**DOI:** 10.1371/journal.pone.0068351

**Published:** 2013-07-08

**Authors:** Kenji Watari, Michio Nakaya, Motohiro Nishida, Kyeong-Man Kim, Hitoshi Kurose

**Affiliations:** 1 Department of Pharmacology and Toxicology, Graduate School of Pharmaceutical Sciences, Kyushu University, Fukuoka, Japan; 2 Pharmacology Laboratory, College of Pharmacy, Chonnam National University, Gwang-Ju, Korea; Virginia Commonwealth University, United States of America

## Abstract

Beta-arrestins (β-arrestin1 and β-arrestin2) are known as cytosolic proteins that mediate desensitization and internalization of activated G protein-coupled receptors. In addition to these functions, β-arrestins have been found to work as adaptor proteins for intracellular signaling pathways. β-arrestin1 and β-arrestin2 are expressed in the heart and are reported to participate in normal cardiac function. However, the physiological and pathological roles of β-arrestin1/2 in myocardial infarction (MI) have not been examined. Here, we demonstrate that β-arrestin2 negatively regulates inflammatory responses of macrophages recruited to the infarct area. β-arrestin2 knockout (KO) mice have higher mortality than wild-type (WT) mice after MI. In infarcted hearts, β-arrestin2 was strongly expressed in infiltrated macrophages. The production of inflammatory cytokines was enhanced in β-arrestin2 KO mice. In addition, p65 phosphorylation in the macrophages from the infarcted hearts of β-arrestin2 KO mice was increased in comparison to that of WT mice. These results suggest that the infiltrated macrophages of β-arrestin2 KO mice induce excessive inflammation at the infarct area. Furthermore, the inflammation in WT mice transplanted with bone marrow cells of β-arrestin2 KO mice is enhanced by MI, which is similar to that in β-arrestin2 KO mice. In contrast, the inflammation after MI in β-arrestin2 KO mice transplanted with bone marrow cells of WT mice is comparable to that in WT mice transplanted with bone marrow cells of WT mice. In summary, our present study demonstrates that β-arrestin2 of infiltrated macrophages negatively regulates inflammation in infarcted hearts, thereby enhancing inflammation when the β-arrestin2 gene is knocked out. β-arrestin2 plays a protective role in MI-induced inflammation.

## Introduction

Beta-arrestins are known as adaptor proteins which mediate desensitization and internalization of G protein-coupled receptors (GPCRs) activated by their own agonists [1]. When GPCRs bind agonists, G protein-coupled receptor kinases (GRKs), which consist of seven homologs, phosphorylate the intracellular serine or threonine residues of GPCRs. This phosphorylation facilitates the recruitment of β-arrestins to the agonist-bound GPCRs. As β-arrestins can inhibit G protein activation by steric hindrance, and bind clathrin and adaptin, GPCRs are consequently desensitized and internalized through clathrin-coated pits. In addition to the regulation of GPCRs, recent studies have revealed that β-arrestins function as signal transducers [2], [3]. For instance, β-arrestins were reported to mediate G protein-independent signaling through GPCRs by biased agonists that selectively activate β-arrestin-mediated pathways [2], [3]. In the heart, several studies have demonstrated that biased agonists of angiotensin II type IA receptor or β-adrenergic receptors induce favorable effects against stresses in the cardiomyocytes [4], [5]. Thus, β-arrestins have been proposed to be potential therapeutic targets for cardiovascular diseases such as heart failure [6], [7]. In addition to being a mediator of biased agonists, β-arrestins have been found to be involved in immunological responses. β-arrestins interact with IκBα and TRAF6, and modulate NF-κB signaling [8]–[10]. The interaction between β-arrestins and IκBα inhibits NF-κB activity induced by inflammatory cytokines. A recent *in vivo* study showed that lipopolysaccharide challenge in β-arrestin2 knockout (KO) mice causes higher mortality than in wild-type (WT) mice, due to the excessive production of inflammatory cytokines [10].

A variety of reports have demonstrated that β-arrestins are involved in normal cardiac functions and the development of cardiac diseases [4], [5], [11]. However, the role of β-arrestins in myocardial infarction (MI) has not been reported yet. Furthermore, the abovementioned reports focused mainly on the role of β-arrestins in cardiomyocytes. In the case of MI, various immune cells infiltrate the infarct area, and exert their own functions to induce cardiac remodeling. β-arrestins play an important role in immune responses [10], [12]. Thus, we speculated that β-arrestins can contribute to cardiac remodeling after MI.

In this study, we investigated the role of β-arrestin2 in MI. We have found that β-arrestin2 was up-regulated in the infarct area. Immunohistochemical analysis and bone marrow (BM) transfer experiments revealed that up-regulation of β-arrestin2 was due to an enhanced recruitment of macrophages derived from BM cells to the infarct area. β-arrestin2 KO mice after MI had higher mortality than WT mice. In β-arrestin2 KO mice, inflammatory responses at the infarct area were enhanced in comparison to WT mice. BM transfer experiments demonstrated that the enhanced inflammatory responses in β-arrestin2 KO mice after MI were caused by an inability of β-arrestin2 to inhibit inflammation of infiltrated macrophages. These results indicate that β-arrestin2 inhibits excessive inflammation of infiltrated macrophages after ischemic injury.

## Results

### Expression of β-arrestin2 is Increased by MI

**Figure 1:**
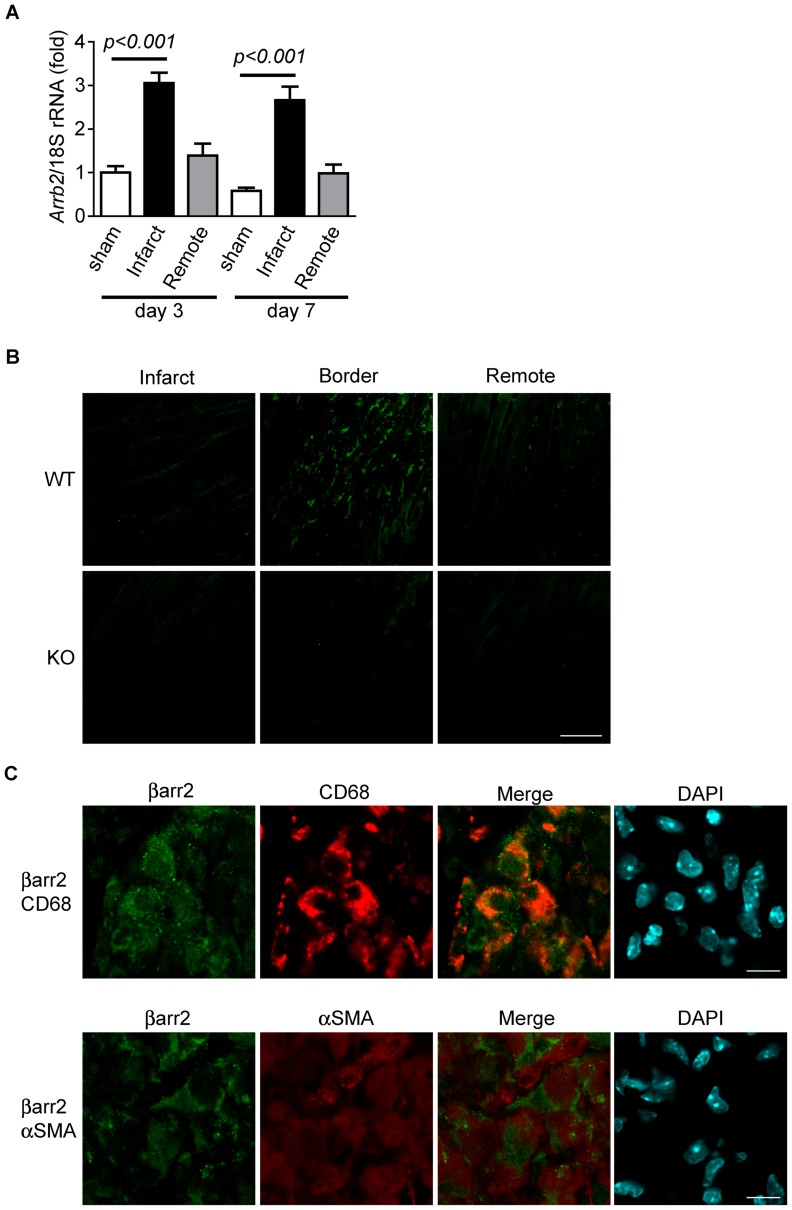
β-arrestin2 is upregulated and highly expressed in infiltrated macrophages after MI. (*A*) Expression of β-arrestin2 mRNA (*Arrb2*) in the infarct and remote areas at the indicated days after MI. Sham-operated ventricles were used as a control. (*B*) Immunohistochemistry of β-arrestin2 in MI-operated hearts. Three days after MI, the cryosections of WT and β-arrestin2 KO hearts were immunostained with anti-β-arrestin2 antibody. Representative images were shown. Three days after MI, the infarct, border, and remote areas of WT and β-arrestin2 KO mice were immunostained with anti-β-arrestin2 antibody. Scale bar, 50 µm. (*C*) Co-immunostaining of anti-β-arrestin2, anti-CD68, or anti-α-SMA antibodies. The hearts of wild-type mice 3 days after MI were immunostained with anti-β-arrestin2, anti-CD68, or anti-α-SMA antibodies. Scale bar, 10 µm.

To assess the role of β-arrestin2 in MI, we first examined the expression of β-arrestin2 mRNA (*Arrb2*) in hearts after MI (permanent occlusion of the left anterior descending artery). A real time reverse-transcription polymerase chain reaction (RT-PCR) experiment demonstrated that β-arrestin2 in the infarct area of left ventricle at 3 and 7 days after MI was increased greatly (Fig. 1A). In contrast, β-arrestin2 expression in the non-infarct remote area of the left ventricle was essentially the same as that in sham-operated mice. The increase was larger at day 3 than at day 7. These results led us to examine what kind of cells are responsible for the increase of β-arrestin2 after MI. Immunohistochemical analysis of heart sections by an anti-β-arrestin2 antibody showed that a strong immunoreactive signal of β-arrestin2 is detected at the border of the infarct area in WT mice (Fig. 1B). On the other hand, the immunoreactive signal at the remote area was almost undetectable. Heart sections from MI-operated β-arrestin2 KO mice were not stained by the anti-β-arrestin2 antibody, which confirmed the specificity of the antibody (Fig. 1B). Then, β-arrestin2 was co-stained with α-SMA or CD68 in MI-operated heart sections. α-SMA and CD68 are marker proteins of myofibroblasts and monocytes/macrophages, respectively. The double-staining experiment demonstrated that the immunoreactive signal of β-arrestin2 was coincident with CD68, but not with α-SMA (Fig. 1C). These results indicate that the up-regulation of β-arrestin2 after MI is due to the recruitment of monocytes/macrophages to the infarct area.

### β-arrestin2 KO Mice Show Increased Mortality after MI

**Figure 2:**
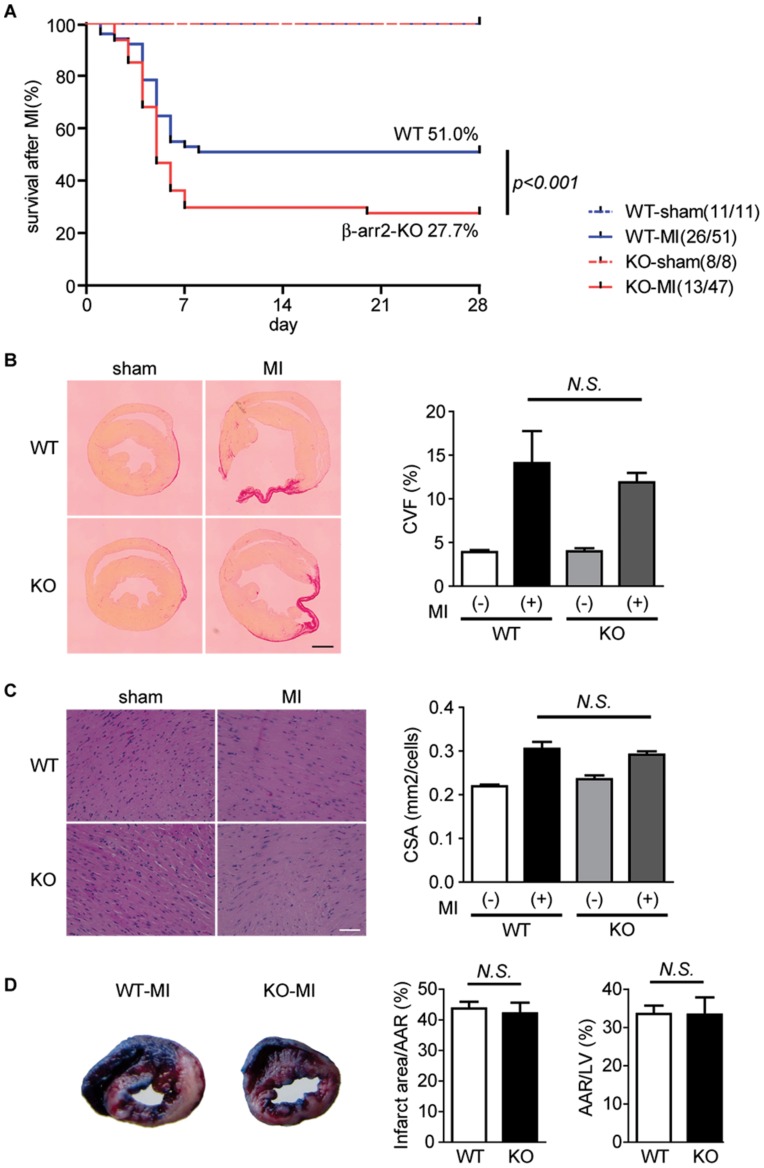
β-arrestin2 KO mice have high mortality in the early phase after MI. (*A*) Kaplan-Meier survival curves of WT (n = 51) and β-arrestin2 KO (n = 47) mice after MI or sham operations. The difference between WT-MI and β-arrestin2 KO-MI was evaluated by the log-rank test (p<0.001). (*B*) Picrosirius red staining of the paraffin-embedded ventricular sections from WT and β-arrestin2 KO mice 28 days after MI. Cross-sections of the ventricles were taken and quantitative data of collagen volume fraction (CVF) was shown in the right panel. The number of mice per group is n = 3 or 4. Scale bar, 1 mm. (*C*) Hematoxylin-eosin (HE) staining of the paraffin-embedded ventricular sections from WT and β-arrestin2 KO mice 28 days after MI. Images were taken at the remote area and cell surface area (CSA) was quantified. The number of mice per group is n = 3 or 4. Scale bar, 50 µm. (*D*) Evans blue dye and 2,3,5-triphenyl tetrazolium chloride (TTC) staining of the heart sections from WT and β-arrestin2 KO mice at 3 hours after coronary ligation. Infarct size and area at risk (AAR) were quantified as a percentage of AAR and left ventricular (LV) area of the section, respectively. The number of mice per group is n = 3.

We next examined the mortality rate of WT and β-arrestin2 KO mice for 28 days after MI. The mice mainly died 4 to 7 days after MI. We found that 25 (49.0%) of 51 WT mice died after MI, while 34 (72.3%) of 47 β-arrestin2 KO mice died (Fig. 2A). Autopsies revealed that the major cause of their death was cardiac rupture. However, there is no difference between WT and β-arrestin2 KO mice in cardiac functions at 3 or 28 days after MI, as assessed by echocardiography and hemodynamic measurements (Tables 1 and 2). Picrosirius red staining and hematoxylin-eosin staining of heart sections 28 days after MI showed that the degree of fibrosis and the extent of hypertrophy were almost the same between WT and β-arrestin2 KO mice (Figs. 2B and C). We further investigated the initial infarct size 3 hours after MI using Evans blue dye and 2,3,5-triphenyl tetrazolium chloride (TTC) staining. The size of area at risk and infarct area was almost same between WT mice and β-arrestin2 KO mice in the initial phase after MI (Fig. 2D). These results indicate that deletion of β-arrestin2 gene leads to high mortality without affecting cardiac functions, fibrosis, hypertrophy, and infarct size after MI.

**Table 1:** Analysis of cardiac functions on day 3 after MI.

	parameters	WT-sham (n = 5)	WT-MI (n = 8)	KO-sham (n = 5)	KO-MI (n = 6)
Organ weight	HW/BW (mg/g)	4.59±0.08	5.93±0.17^###^	4.48±0.08	5.83±0.10^###^
	LW/BW (mg/g)	6.18±0.23	6.62±0.21	6.14±0.12	6.96±0.25
Echocardiography	IVSTd (mm)	0.96±0.02	0.66±0.03^###^	0.92±0.06	0.60±0.00^###^
	LVIDd (mm)	2.60±0.07	3.60±0.14^###^	2.56±0.09	3.28±0.22^##^
	LVPWd (mm)	1.04±0.05	1.29±0.09#	1.00±0.06	1.38±0.08#
	LVIDs (mm)	0.74±0.02	2.85±0.17^###^	0.76±0.06	2.73±0.25^###^
	%EF	97.0±0.0	48.3±4.2^###^	97.6±0.2	41.5±5.4^###^
	%FS	70.2±0.4	21.0±2.2^###^	70.8±1.2	17.3±2.7^###^

HW: heart weight, BW: body weight, LW: lung weight, IVSTd: interventricular septal thickness diastolic, LVIDd: LV inner diameter diastolic, LVPWd: LV posterior wall diameter diastolic, LVIDs: LV inner diameter systolic, %EF: percent ejection fraction, %FS: percent fractional shortening.

# : p<0.05, ^##^: p<0.01, ^###^: p<0.001 vs WT-sham.

**Table 2:** Analysis of cardiac functions on day 28 after MI.

	parameters	WT-sham (n = 13)	WT-MI (n = 16)	KO-sham (n = 8)	KO-MI (n = 9)
Organ weight	HW/BW (mg/g)	4.48±0.05	6.08±0.15^###^	4.59±0.10	6.24±0.23^###^
	LW/BW (mg/g)	5.66±0.09	6.35±0.32	5.58±0.26	7.07±0.57
Echocardiography	IVSTd (mm)	1.05±0.03	0.73±0.05^###^	1.08±0.05	0.65±0.05^###^
	LVIDd (mm)	2.90±0.08	4.17±0.13^###^	2.96±0.07	4.57±0.27^###^
	LVPWd (mm)	1.27±0.07	1.91±0.08^###^	1.35±0.08	2.04±0.08^###^
	LVIDs (mm)	0.85±0.05	3.40±0.14^###^	1.01±0.11	3.96±0.27^###,§^
	%EF	96.4±0.8	44.2±2.4^###^	95.1±1.5	33.1±2.6^###,§§§^
	%FS	69.4±1.8	18.8±1.3^###^	66.1±2.8	13.5±1.1^###,§^
Hemodynamic analysis	HR (bpm)	503±1.3	496±0.7	500±1.1	496±0.9
	dp/dt_max_ (mmHg/s)	11134±478	7940±301^###^	12312±722	7403±444^###^
	dp/dt_min_ (mmHg/s)	6954±149	5201±199^###^	8253±299^###^	4832±256^###^
	EDP (mmHg)	0.21±0.96	9.49±1.16^###^	3.94±1.73	13.58±2.73^###^
	Tau (s)	11.2±0.5	18.4±1.5^###^	10.6±0.3	19.1±1.7^##^

HW: heart weight, BW: body weight, LW: lung weight, IVSTd: interventricular septal thickness diastolic, LVIDd: LV inner diameter diastolic, LVPWd: LV posterior wall diameter diastolic, LVIDs: LV inner diameter systolic, %EF: percent ejection fraction, %FS: percent fractional shortening. HR: heart rate, EDP: end-diastolic pressure. ^##^: p<0.01, ^###^: p<0.001 vs WT-sham. ^§^: p<0.05, ^§§§^: p<0.001 vs WT-MI.

### Inflammation is Enhanced in β-arrestin2 KO Mice after MI

**Figure 3:**
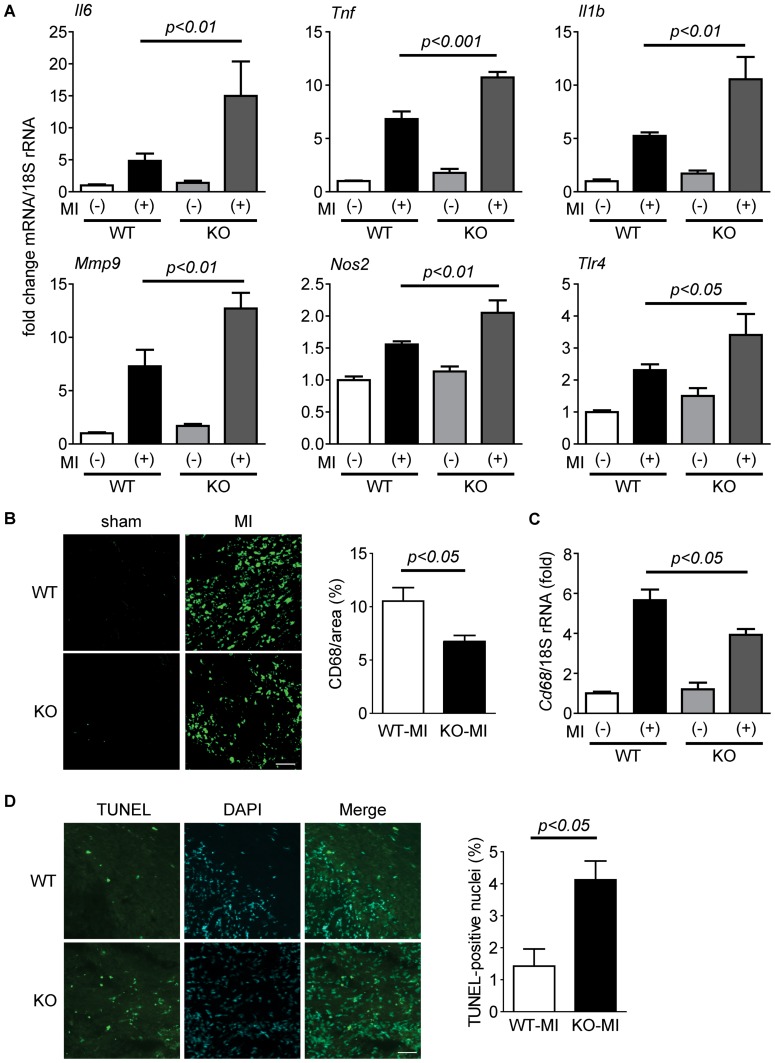
Inflammatory responses in β-arrestin2 KO mice are enhanced in the early phase after MI. (*A*) mRNA expression levels of pro-inflammatory genes in the hearts of WT and β-arrestin2 KO mice at 3 days after MI. Total RNA extracted from sham-operated ventricles or MI-operated infarct area were subjected to real time RT-PCR. WT-sham: n = 3, KO-sham: n = 4, WT-MI: n = 5, KO-MI: n = 3. (*B*) Infiltration of CD68-positive monocytes/macrophages into the infarct area (n = 4–6) in WT and β-arrestin2 KO mice. Representative results were shown. Quantitative data were shown in the right panel. (*C*) mRNA expression of CD68 (*Cd68*) in the infarcted area of WT and β-arrestin2 KO mice at 3 days after MI. (*D*) TUNEL-positive nuclei at the border zone (WT-MI: n = 3, KO-MI: n = 5) of WT and β-arrestin2 KO mice at 3 days after MI. Scale bar, 50 µm.

Myocardial infarction strongly induces inflammation at the infarct area [13]. Therefore, we examined the expression levels of inflammatory genes in the heart of β-arrestin2 KO mice after MI. A quantitative RT-PCR experiment revealed that several inflammatory genes, such as TNF-α (*Tnf*) and IL-6 (*Il6*), had markedly increased in β-arrestin2 KO mice when compared to WT mice 3 days after MI (Fig. 3A). We also measured mRNA expression of anti-inflammatory cytokines, such as TGF-β (*Tgfb1*) and IL-10 (*Il10*). TGF-β was increased in β-arrestin2 KO mice after MI, while IL-10 was not significantly changed (Fig. S1). Then, we examined the degree of infiltration of monocytes/macrophages after MI in β-arrestin2 KO mice. CD68 is a marker of monocytes/macrophages, and CD68 staining demonstrated that the infiltration of monocytes/macrophages is reduced in β-arrestin2 KO mice when compared to WT mice (Fig. 3B). This result is consistent with the analysis of quantitative RT-PCR data indicating expression level of CD68 mRNA (*Cd68*) in the infarct area of hearts was lower in MI-operated β-arrestin2 KO mice than in MI-operated WT mice (Fig. 3C). On the other hand, there were no significant differences in the number of bone marrow cells (Fig. S2A) or cell growth and differentiation (Fig. S2B) between bone marrow (BM)-derived macrophages from WT and β-arrestin2 KO mice. These results rule out the possibility that the increased expression of inflammatory genes in MI-operated β-arrestin2 KO mice was due to an increase in the number of infiltrated macrophages at the infarct area. Our detailed analysis revealed that M2 macrophages [14] appeared in the infarct area 3 days after MI (Fig. S3A). In addition, quantitative RT-PCR demonstrated that the number of the M2 macrophages were higher in β-arrestin2 KO mice than in WT mice after MI (Fig. S3B). As it has been reported that excessive inflammation could lead to tissue damage [15], [16], we next performed terminal deoxynucleotidyl transferase-mediated dUTP nick end-labeling (TUNEL) staining, which detects DNA fragmentation accompanied by apoptosis. At the border zone of the infarct area, more TUNEL-positive nuclei were detected in β-arrestin2 KO mice than in WT mice (Fig. 3D). These results indicate that excessive inflammation in β-arrestin2 KO mice lead to increased number of apoptotic cells.

### β-arrestin2 in Infiltrated Macrophages Prevents Excessive Inflammatory Responses

**Figure 4:**
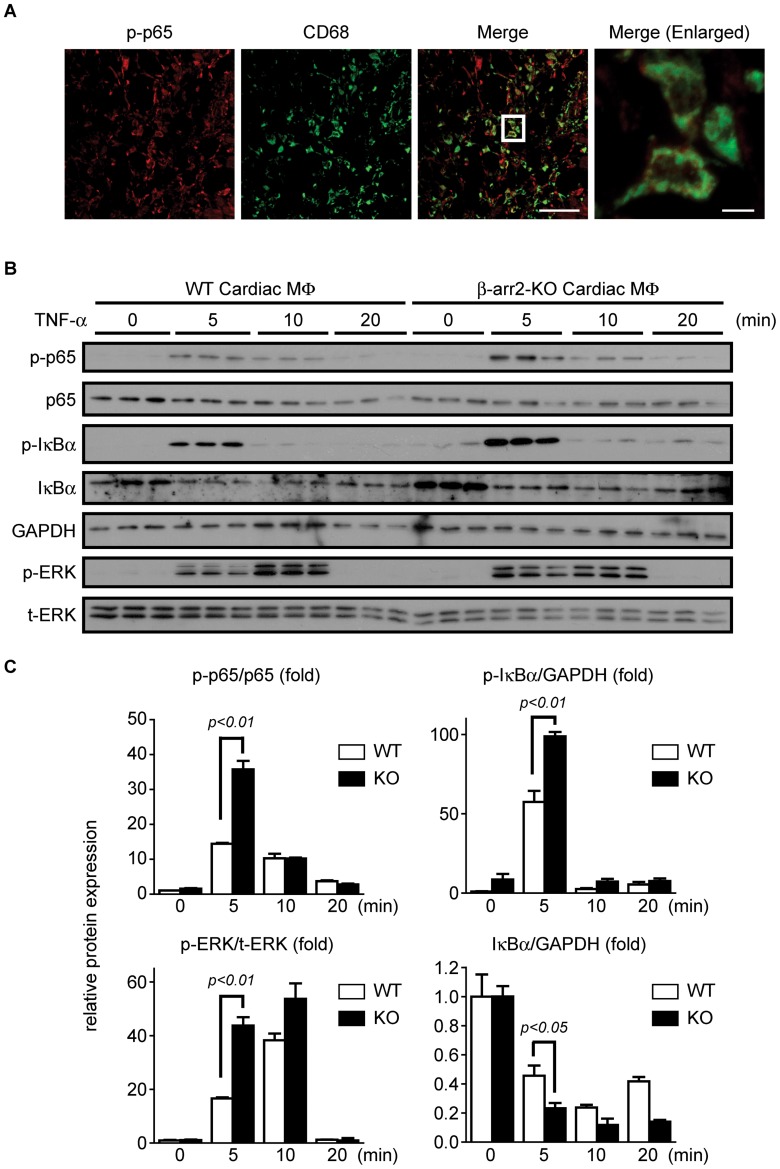
NF-κB signaling of cardiac macrophages is enhanced in β-arrestin2 KO mice by MI. (*A*) Heart section of WT mice at 3 days after MI were stained with phospho-p65 (p-p65) (red) and CD68 (green). Enlarged images are shown on the fourth column. Scale bar, Merge: 50 µm, Merge (Enlarged): 5 µm. (*B*, *C*) Cardiac macrophages from WT and β-arrestin2 KO mice were stimulated with mouse TNF-α (10 ng/mL) at the indicated length of time. (*B*) Samples were loaded on SDS-PAGE and subjected to Western blot using the antibodies indicated. Phospho-p65 (p-p65) and phospho-ERK (p-ERK) were normalized with p65 and total-ERK, respectively. GAPDH was used for normalization of IκBα and phospho-IκBα (p-IκBα). (*C*) Densitometric analysis of the Western blot. White columns or black columns represent data obtained from cardiac macrophages of WT mice and β-arrestin2 KO mice.

β-arrestin2 is reported to modulate NF-κB signaling that regulates expression of inflammatory genes [8]–[10]. Therefore, we first examined the activation of NF-κB signaling in the infarct area. Immunohistochemical study revealed that p65, one of components of NF-κB, was phosphorylated at the border zone of MI-operated heart (Fig. 4A). This p65 phosphorylation signal was mainly detected in CD68-positive infiltrated macrophages. In contrast, p65 phosphorylation was not observed in CD31-positive endothelial cells and α-actinin-positive cardiomyocytes (Figs. S4A, S4B). These data demonstrate that NF-κB signaling is primarily activated in the macrophages at the infarct area. As β-arrestin2 was strongly expressed in monocytes/macrophages infiltrating the infarct area, we hypothesized that deletion of β-arrestin2 in the monocytes/macrophages causes excessive inflammation through NF-κB signaling after MI. Then, we isolated macrophages from the hearts of WT or β-arrestin2 KO mice 3 days after MI. In our isolation protocol, about 85% of isolated cells were CD11b-positive, which is a marker of myeloid cells (Fig. S5). This result indicated that the isolated cells mainly consist of macrophages. The percentage of isolated CD11b-positive cells was almost the same between WT and β-arrestin2 KO mice (Fig. S5). Treatment of the macrophages with TNF-α induced phosphorylation of p65 (Figs. 4B, 4C). Notably, p65 phosphorylation was strongly enhanced in the macrophages taken from β-arrestin2 KO mice. TNF-α stimulates not only p65 phosphorylation but also IκBα phosphorylation and ERK activation [17], [18]. We found that IκBα and ERK were also strongly phosphorylated by TNF-α treatment of the macrophages from β-arrestin2 KO mice (Figs. 4B, 4C). Consistent with the result related to IκBα phosphorylation, IκBα after TNF-α stimulation was degraded more rapidly in macrophages from β-arrestin2 KO mice than that from WT mice (Figs. 4B, 4C). These results indicate that β-arrestin2 in infiltrated macrophages plays an important role in the prevention of excessive inflammation at the infarct area after MI by negatively modulating NF-κB signaling.

### Wild-type Mice Transplanted with β-arrestin2 KO Bone Marrow Cells Show Enhanced Inflammatory Responses after MI

**Figure 5:**
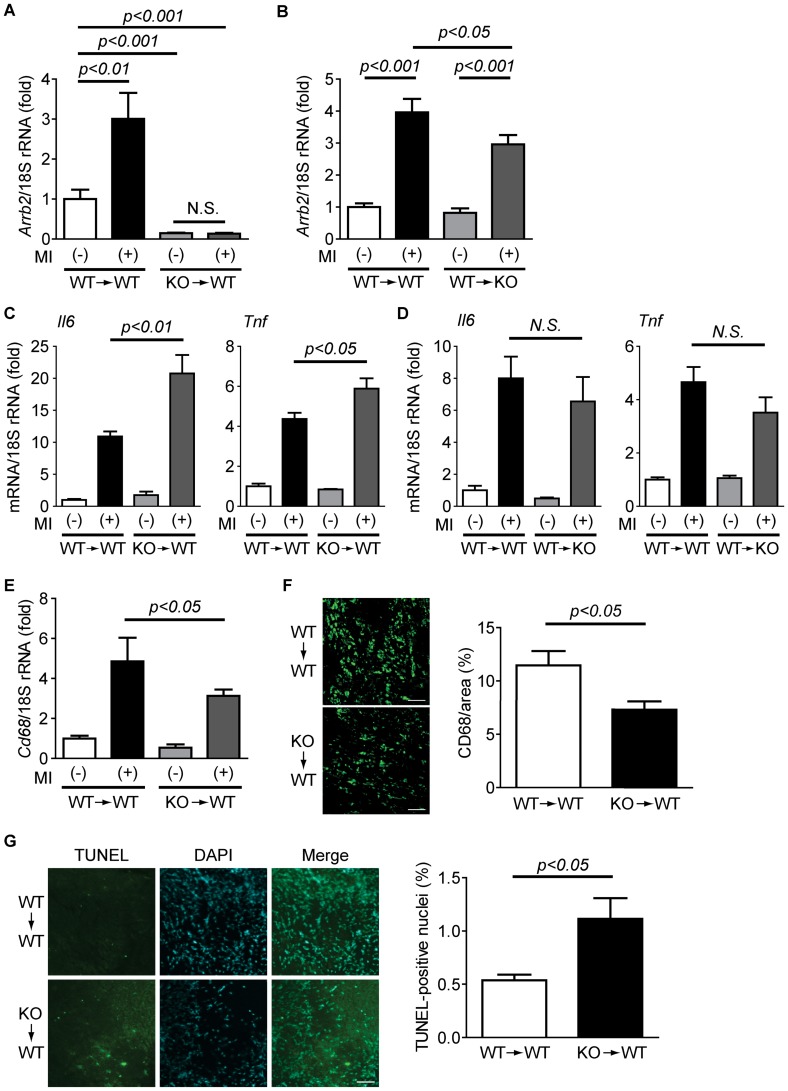
BM cells from β-arrestin2 KO mice enhance the inflammatory responses after MI. (*A*, *B*) Expression of β-arrestin2 mRNA (*Arrb2*) 3 days after MI of BM-transplanted mice. (*A*) WT mice were transplanted with BM cells from WT or β-arrestin2 KO mice. WT → WT sham: n = 3, WT → WT MI: n = 3, KO → WT sham: n = 3, KO → WT MI: n = 5. (*B*) WT or β-arrestin2 KO mice were transplanted with BM cells from WT mice. Sham-operated heart was used as a control. WT → WT and WT → KO: n = 6. Three days after MI, the expression of *Arrb2* was determined in the heart. (*C*, *D*) mRNA expression levels of inflammatory genes 3 days after MI in the heart of BM-transplanted mice. (*E*) mRNA expression of *Cd68* at 3 days after MI in the heart of WT mice received BM cells from WT (WT → WT) or β-arrestin2 KO (KO → WT) mice. (*F*) Infiltration of CD68-positive monocytes/macrophages into the infarcted hearts. (*G*) TUNEL-positive nuclei at the border zone of the hearts in WT mice reconstituted with BM cells from WT or β-arrestin2 KO mice 3 days after MI. Scale bar, 50 µm.

To further clarify the role of β-arrestin2 in the inflammation at the infarct area, we performed bone marrow (BM) transfer experiments. Lethally-irradiated WT mice were reconstituted with BM cells from WT or β-arrestin2 KO mice. The average reconstitution efficiency evaluated by FACS analysis of peripheral blood was 97.2%. We then performed MI with these BM-transferred mice. In mice transplanted with BM cells from WT mice (WT → WT), β-arrestin2 mRNA (*Arrb2*) at the infarct area was increased by MI (Fig. 5A). In contrast, in mice transplanted with BM cells from β-arrestin2 KO mice (KO → WT), the up-regulation of β-arrestin2 mRNA was abolished (Fig. 5A). Furthermore, the expression level of β-arrestin2 mRNA was extremely low in the sham group transplanted with BM cells from β-arrestin2 KO mice. To evaluate the contribution of BM-derived cells to the expression of β-arrestin2 in the heart after MI more closely, we further performed BM transplantation from WT mice to WT or β-arrestin2 KO mice (WT → WT, or WT → KO, respectively). The expression of β-arrestin2 in WT → KO mice at the infarct area was increased, reaching comparable levels to that in WT → WT mice after MI (Fig. 5B). These results indicate that BM-derived cells are responsible for the expression of β-arrestin2 in the heart. The expression levels of inflammatory genes, such as IL-6 (*Il6*) and TNF-α (*Tnf*), were significantly enhanced by MI in mice transplanted with BM cells from β-arrestin2 KO mice (KO → WT) when compared to mice transplanted with BM cells from WT mice (WT → WT) (Fig. 5C). In contrast, the expression level of inflammatory genes in the infarct area of β-arrestin2 KO mice transplanted with BM cells from WT mice (WT → KO) was comparable to that of WT → WT mice (Fig. 5D). Despite the up-regulation of inflammatory genes in WT mice transplanted with β-arrestin2 KO mice (KO → WT), CD68 mRNA (*Cd68*) is down-regulated in KO → WT mice (Fig. 5E). Moreover, immunohistochemical analysis of CD68 showed that infiltration of the macrophages was reduced in mice transplanted with BM cells from β-arrestin2 KO mice (KO → WT), indicating that the macrophages of β-arrestin2 KO mice have a defect in their migration ability (Fig. 5F). In addition to the lower infiltration of macrophages, the number of TUNEL-positive nuclei was increased in mice transplanted with BM cells from β-arrestin2 KO mice (KO → WT) (Fig. 5G). These results indicate that β-arrestin2 in the macrophages negatively regulates the expression of inflammatory genes, and the enhanced inflammation in β-arrestin2 KO mice leads to apoptosis of cardiac cells.

## Discussion

In this report, we demonstrated that β-arrestin2 protects the cells against excessive inflammation after MI. β-arrestin2 expression in the heart was increased by MI. This increase was caused by the recruitment of macrophages to the infarct area. In that area, the production of inflammatory genes was enhanced in β-arrestin2 KO mice in comparison with WT mice. Several reports have shown that the production of inflammatory genes is regulated by NF-κB signaling complex [15], [18], [19]. We found that phosphorylation of p65 was increased in CD68-positive infiltrated macrophages in the heart. Furthermore, TNF-α stimulation of infiltrated macrophages resulted in enhanced phosphorylation of p65 in β-arrestin2 KO mice when compared to WT mice. Therefore, we assumed that MI enhances NF-κB activity in the infiltrated macrophages of β-arrestin2 KO mice, and leads to the excessive induction of inflammatory genes. Consistent with enhanced inflammation, more apoptotic cells were found in the hearts of β-arrestin2 KO mice in comparison to those of WT mice. By BM transplantation experiments from β-arrestin2 KO to WT mice, we found that β-arrestin2 of infiltrated macrophages modulates enhanced inflammatory responses by MI. Results of BM transplantation from WT to β-arrestin2 KO mice also supported the finding.

Three days after MI, expression levels of several inflammatory genes, such as IL-6 and TNF-α, were increased in WT and β-arrestin2 KO mice. The degree of increase was higher in β-arrestin2 KO mice than in WT mice. As inflammatory cytokines are reported to induce cardiomyocyte apoptosis [19], [20], it is possible that the enhanced apoptosis in the infarct area of β-arrestin2 KO mice is caused in part by the increased production of TNF-α from infiltrated macrophages. It has also been reported that β-arrestin2 inhibits the cell apoptosis [21], [22]. Thus, β-arrestin2 in infiltrated macrophages could inhibit the excessive inflammation after MI (Figs. 5C, 5D), and in cardiomyocytes prevent the cardiomyocyte apoptosis from ischemic injury. Further studies are necessary to verify the contribution of β-arrestin2 in non-hematopoietic cardiac cells to the pathology after MI.

We found that the expression level of β-arrestin2 increases during MI. This increase is mainly due to the recruitment of macrophages from BM to the infarct areas. Although cardiomyocytes and fibroblasts express β-arrestin2, their expression levels are low when compared to that of infiltrated macrophages. It is possible that β-arrestin2 in cardiomyocytes and fibroblasts plays a role in physiological and pathological responses during MI. However, β-arrestin2 in these cells does not contribute to inflammation (Fig. 5D). Increased expression of β-arrestin2 may have a physiological function. β-arrestin2 expression could be increased by inflammatory cytokines to prevent excess inflammation, as β-arrestin2 protects the cells against inflammation and prevents excess inflammation. As we did not measure the expression level of β-arrestin2 in BM, we do not know whether β-arrestin2 in infiltrated macrophages is increased by inflammation. It would be interesting to compare the expression level of β-arrestin2 between BM cells and infiltrated macrophages.

The macrophages recruited to the infarct area strongly expressed β-arrestin2. However, the number of macrophages that infiltrated into the infarct area was decreased in β-arrestin2 KO mice. β-arrestin2 has been reported to regulate the chemotaxis by regulating actin assembly and the contribution of β-arrestin2 to the chemotaxis is thought to be dependent on the cell type, inflammatory environment, and receptors [23]. In our study, β-arrestin2 in the macrophages positively regulates the macrophage chemotaxis, consistent with a previous report showing the defective lymphocyte chemotaxis in β-arrestin2 KO mice [24]. In spite of the decrease in the number of the recruited macrophages, inflammatory responses were enhanced at the infarct area in β-arrestin2 KO mice. Our bone marrow transplantation experiment revealed that β-arrestin2 expression in infiltrated macrophages is essential for inhibiting inflammatory responses after MI (Fig. 5C), although β-arrestin2 is reported to express in other types of cells, such as cardiomyocytes and cardiac fibroblasts [25]. The immunological function of β-arrestin2 seemed to modulate the NF-κB signaling by the interaction with IκBα (Fig. 4B) [8], [9]. These results suggest that the infiltrated macrophages are mainly responsible for the inflammatory responses at the infarct area and excessive inflammatory responses of β-arrestin2 KO mice are evoked in each infiltrated β-arrestin2 KO macrophage. Taken together, we propose that β-arrestin2 in infiltrated macrophages plays two different roles in chemotaxis and inflammation at the infarct area.

Previous reports demonstrated that β-arrestin2 negatively regulates the activity of NF-κB by modulating IκBα degradation [8], [9], lipopolysaccharide-induced endotoxin shock [10], and sepsis-induced inflammation [12]. Consistent with these reports, we found that TNF-α stimulation induced phosphorylation of p65 in macrophages isolated from the infarct area, and the degree of phosphorylation was higher in β-arrestin2 KO mice than in WT mice. This suggests that β-arrestin2 negatively regulates NF-κB signaling in infiltrated macrophages. Breakdown of this negative regulation of β-arrestin2 in NF-κB signaling results in the increased expression of inflammatory genes. Among the inflammatory genes regulated by NF-κB, increased expression of IL-6 in β-arrestin2 KO mice is intriguing, as IL-6 is reported to increase the rate of death after MI [26]. Furthermore, IL-6 stimulation increases the production of IL-6 and other cytokines [27]. Positive feedback of IL-6 may lead to excessive inflammation in the early phase after MI.

To date, it has been suggested that macrophages are recruited to infectious or damaged site and play important roles for healing the pathological conditions [28]. Infiltrated macrophages are largely classified in two types, M1 and M2 macrophage, respectively [29]. It has recently been proposed that macrophages infiltrating into injured heart after MI have different properties in different phases [14] and macrophage transition, M1 to M2 is critical for the survival after MI [30], [31]. However, precise roles of M1 and M2 macrophages in the MI are poorly understood. We found that the numbers of M2 macrophages were higher in the hearts of β-arrestin2 KO mice than that of WT mice on day 3 after MI (Fig. S3B). β-arrestin2 may be involved in the M2 transition. It would be intriguing to examine the role of β-arrestin2 in M2 polarization *in vitro*.

We found that the expression levels of β-arrestin2 were markedly reduced in the hearts of WT mice transplanted with BM cells from β-arrestin2 KO mice. As resident macrophages are a rare population in non-infarcted hearts (Fig. 3B), this result indicates that some portion of cardiac macrophages is continuously replaced by BM-derived cells. To verify the role of resident macrophages in normal cardiac functions and inflammation, identification of the cell population may provide clarification. BM cells are differentiated into fibrocytes that share some similarities with macrophages and that play an important role in chronic inflammation [32]. As β-arrestin2 is involved in inflammatory responses in macrophages, it would be interesting to examine whether fibrocytes express β-arrestin2 and whether β-arrestin2 affects inflammatory responses of fibrocytes.

Taken together, we demonstrated the role of β-arrestin2 in MI. β-arrestin2 was highly expressed in infiltrated macrophages, and negatively regulates apoptosis and excessive inflammation after MI. β-arrestin2 KO mice showed high mortality in the early phase after MI. Emerging evidence has revealed the importance of β-arrestin2-mediated signaling in heart failure [6], [7]. For instance, β-arrestin2 is reported to protect the heart against detrimental effects by excessive β-adrenergic receptor stimulation during heart failure [5]. Although most reports have focused on the role of β-arrestin2 in cardiomyocytes, our study clearly shows that the expression level of β-arrestin2 in infiltrated macrophages is higher than that in cardiomyocytes and fibroblasts, and β-arrestin2 inhibits inflammatory responses by modulating IκBα degradation. Our study provides new insight into the role of β-arrestin2 in the diseased state of the heart.

## Materials and Methods

### Surgical Procedures of MI

We purchased C57BL/6J mice from Kyudo (Japan). β-arrestin2 KO mice were obtained by Dr. R. J. Lefkowitz (Duke University). Male mice (8–10 weeks old) were subjected to permanent left anterior descending coronary artery ligation in anesthetized condition (50 mg/kg sodium pentobarbital). The sham-operated animals underwent the same procedure without ligation of the coronary artery. All animal experiments were approved by Animal Care and Use Committee, Kyushu University.

### Echocardiographic Analysis and Hemodynamic Measurement

Echocardiographic and hemodynamic parameters are measured as described [33]. We performed transthoracic ultrasound cardiography at 3 and 28 days after MI under anesthesia. Two-dimensional targeted M-mode tracings were recorded using Nemio GX image analyzing system (SSA-580A, Toshiba Medical Systems). Percent fractional shortening (%FS) was calculated as %FS =  [(LVIDd-LVIDs)/LVIDd] ×100 to estimate the cardiac systolic function. We conducted hemodynamic measurement 28 days after MI with a micromanometer and conductance 1.4 F catheter (Millar Instruments). Under anesthesia, a catheter was inserted into left ventricles. The signals were analyzed by Digi-Med Heart Performance Analyzer (Micro-Med) and Digi-Med System Integrator (Micro-Med).

### Antibodies

The antibodies are purchased from Cell Signaling (IκBα, β-arrestin2, phospho-ERK, phospho-IκBα and phospho-p65), Santa Cruz (IκBα, GAPDH, p65 and Arginase-1), BD Pharmingen (CD31), Sigma-Aldrich (α-actinin), Promega (ERK), AbD Serotec (CD68), BioLegend (CD11b and CD16/32), and Thermo Scientific (α-SMA). These antibodies were used for immunoblotting, immunohistochemistry, and flow cytometry.

### Histological Analysis

The paraffin-embedded ventricular sections (5 µm in thickness) were stained with picrosirius red and hematoxylin-eosin staining and observed under microscopy (KEYENCE BZ-9000). Collagen volume fraction of ventricular sections and cross sectional area at the remote region was calculated using BZ-II analyzer (Keyence). Cryostat sections (4 µm in thickness) embedded in FSC22 Frozen Section Media (Leica Microsystems) were used for immunohistochemistry. The sections cut from the heart were air-dried, and fixed in acetone at 4°C for 10 min. Reaction with primary anti-mouse CD68 was performed overnight at 4°C. The images were quantified and averaged from 5 randomly selected fields (x 40). We used Metamorph analysis software for quantitative analyses. For colocalization study, excised hearts were fixed in 4% paraformaldehyde overnight and embedded in FSC22 Frozen Section Media. Sections (4 µm in thickness) were immunostained with antibodies recognizing β-arrestin2, CD68, phospho-p65, Arginase-1, α-SMA, CD31, and α-actinin.

### Analysis of Apoptosis

To detect apoptosis, cryostat sections (4 µm in thickness) fixed in acetone at 4°C for 10 min were subjected to TUNEL staining (Millipore) according to the manufacturer’s instruction. The images were quantified and averaged from 5 randomly selected fields (x 40). The number of TUNEL-positive nuclei was counted by using Metamorph analysis software. Nuclei were identified with DAPI (4',6-diamino-2-phenylindole) and the data were normalized per total nuclei in the same sections.

### Assessment of Infarct Size

At 3 hours after coronary ligation, euthanized mice were perfused with 1% Evans blue dye and their hearts were excised. 1 mm thick sections of the left ventricles were immediately prepared, and put in 1% solution of 2,3,5-triphenyl tetrazolium chloride (TTC) in phosphate buffer saline (PBS) for 30 min at 37°C. We put these sections overnight in 10% neutral buffered formalin. Sections were digitally photographed using a digital camera and quantified by Adobe Photoshop 7.0 software. Necrotic area (TTC-negative) was shown as a percentage of area at risk (AAR; area not stained with Evans blue dye) and AAR was shown as a percentage of the left ventricular area as previously described [34].

### BM Transplantation

BM transfer experiments were performed according to the previous report. WT or β-arrestin2 KO male mice of 6 weeks old were lethally irradiated with 10 Gy from cesium gamma source. WT or β-arrestin2 KO male mice of 6 weeks old were served as BM donor. BM cells were collected from femurs and tibias of donor mice. BM cells were resuspended in phosphate-buffered saline. Each recipient mouse was injected with BM cells through the orbital vein. BM cells from a mouse were typically used for 8–10 gamma-irradiated mice. Four weeks after transplantation, these mice were subjected to MI.

### Preparation of BM-derived Macrophages

BM cells were collected from femurs and tibias of WT or β-arrestin2 KO mice, and plated on the 6cm non-treated dishes in MEM Alpha (Invitrogen) supplemented with 10% fetal bovine serum (FBS) and macrophage colony-stimulating factor (M-CSF) at a density of 1×10^5^ cells/mL. Three or 6 days after plating, cells were once washed with phosphate-buffered saline (PBS) and detached with 0.02% EDTA (Nacalai Tesque) for counting the number of attached cells.

### mRNA Expression Analysis

Total RNA was extracted from sham- or MI-operated hearts using ISOGEN (Nippon Gene) and purified with RNeasy Fibrous Tissue Mini Kit (QIAGEN). mRNA expression was quantified by real time RT-PCR using TaqMan probes. Specific primers used in this study are shown in Table 3. TaqMan probes for mouse *Arrb2* (Assay ID: Mm00520665_m1) and mouse *Arg1* (Assay ID: Mm00475988_m1) were purchased from Applied Biosystems. Data were normalized to 18S rRNA.

**Table 3:** Sequences of primers used for real time RT-PCR.

mRNAs	primers
*Tnf*	Forward: 5′- GTTCTCTTCAAGGGACAAGGCTG -3′
	Reverse: 5′- TCCTGGTATGAGATAGCAAATCGG -3′
	probe: 5′- TACGTGCTCCTCACCCACACCGTCA -3′
*Il1b*	Forward: 5′- GAAAGACGGCACACCCACC -3
	Reverse: 5′- AGACAAACCGCTTTTCCATCTTC -3′
	probe: 5′- TGCAGCTGGAGAGTGTGGATCCCAA -3′
*Il6*	Forward: 5′- GGGACTGATGCTGGTGACAA -3′
	Reverse: 5′- TGCCATTGCACAACTCTTTTCT -3′
	probe: 5′- TCACAGAGGATACCACTCCCAACAGACCTG -3′
*Mmp9*	Forward: 5′- GGCCCCAGGAGTCTGGATA -3′
	Reverse: 5′- AATAGGCTTTGTCTTGGTACTGGAA -3′
	probe: 5′- ACCCACGTCAGCGGGCTTCTCC -3′
*Nos2*	Forward: 5′- ACATCAGGTCGGCCATCACT -3′
	Reverse: 5′- CGTACCGGATGAGCTGTGAAT -3′
	probe: 5′- CCCCCAGCGGAGTGACGGC -3′
*Tlr4*	Forward: 5′- AAACTTGCCTTCAAAACCTGGC -3′
	Reverse: 5′- ACCTGAACTCATCAATGGTCACATC -3′
	probe: 5′- CACGTCCATCGGTTGATCTTGGGAGAA -3′
*Cd68*	Forward: 5′- CTGCTGTGGAAATGCAAGCATA -3′
	Reverse: 5′- CCCGAAGTGTCCCTTGTCA -3′
	probe: 5′- TCTCTCTAAGGCTACAGGCTGCTCAGCTGC -3′
*Tgfb1*	Forward: 5′- AATTCCTGGCGTTACCTTGGT -3′
	Reverse: 5′- TGTATTCCGTCTCCTTGGTTCA -3′
	probe: 5′- CCGGCTGCTGACCCCCACTGATA -3′
*Il10*	Forward: 5′- TTTGAATTCCCTGGGTGAGAAG -3′
	Reverse: 5′- CTCCACTGCCTTGCTCTTATTTTC -3′
	probe: 5′- AGGCGCTGTCATCGATTTCTCCCC -3′

### MI-induced Cardiac Macrophages

To prepare cell suspension of the macrophages from infarcted heart, MI-operated hearts were excised from WT or β-arrestin2 KO mice, and minced with fine scissors. Minced hearts were placed into a cocktail of 2.5 mg/ml trypsin, 4.4 mg/ml Dispase II (Sigma-Aldrich), and 1 mM EDTA in phosphate-buffered saline, and shaken at 37°C for 10 min, followed by a centrifugation (300×g, 4 min). The cell pellet was suspended in DMEM +10% fetal bovine serum (FBS). The procedures of suspension and centrifugation were repeated 3 times. The cells were then collected and 2×10^5^ cells were cultured in non-treated 24 wells dishes. One day later, cells were starved with DMEM supplemented with 0.5% heat-inactivated FBS for 10 h and stimulated with mouse TNF-α (10 ng/mL, PeproTech). Cells were then lysed with lysis buffer containing 50 mM Tris-Cl (pH 7.5), 1 mM EDTA, 150 mM NaCl, 10% glycerol, 20 mM NaF, 0.5% Nonidet P-40, supplemented with 1% protease inhibitor cocktail, and PhosSTOP. After centrifugation, supernatants were collected and eluted with SDS sample buffer. The samples were loaded on SDS-PAGE, followed by Western blot. Densitometric analysis was performed using ImageJ software.

### Flow Cytometry

Cardiac macrophages were suspended in phosphate-buffered saline containing 2% heat-inactivated FBS and 0.05% NaN_3_. For surface staining of CD11b, cells were pretreated with anti-CD16/32 on ice for 10 min, and then treated with FITC-conjugated anti-CD11b on ice for 60 min. Stained cells were washed and were analyzed with a FACSCalibur (BD Biosciences).

### Statistical Analysis

The results are presented as means ± SEM from at least three independent experiments. Statistical analysis was performed by two-tailed Student’s t-test (for 2 groups comparison) or one-way Analysis of Variance followed by Student-Neuman-Keuls procedure (for multiple groups comparison).

## Supporting Information

Figure S1 **mRNA expression levels of anti-inflammatory genes, TGF-β1 (** ***Tgfb1*** **) and IL-10 (** ***Il10*** **), in the hearts of WT and β-arrestin2 KO mice at 3 days after MI.** Total RNA extracted from sham-operated ventricles or MI-operated infarct area were subjected to real time RT-PCR.
(TIF)

Figure S2 **Numbers of bone marrow (BM) cells collected from a femur of WT and β-arrestin2 KO mice (** ***A*** **).** n = 3 per each group. (*B*) Differentiation of BM-derived cells from WT and β-arrestin2 KO mice into BM-derived macrophages. BM-derived cells plated on the non-treated dishes were cultured in the α-MEM containing 10% FBS, and macrophage colony-stimulating factor on 6 cm plate. They were detached and the number of BM-derived cells was counted at 3 or 6 days after plating. n = 4 per each group.
(TIF)

Figure S3 **Immunohistochemical analysis of the expression of Arginase-1 on the heart section of MI-operated WT mice (** ***A*** **).** CD68 and DAPI were used as markers of monocytes/macrophages and cell nuclei, respectively. Scale bar, 50 µm. (*B*) mRNA expression level of Arginase-1 (*Arg1*) 3 days after MI in the heart of WT and β-arrestin2 KO mice.
(TIF)

Figure S4 **Immunohistochemical analysis of p65 phosphorylation in endothelial cells and cardiomyocytes.** Phospho-p65 (p-p65) (red) was co-stained with (*A*) CD31 or (*B*) α-actinin (green) at the infarct area on the heart section of WT mice at 3 days after MI. Scale bar, 50 µm.
(TIF)

Figure S5 **Flow cytometry of cardiac macrophages from WT and β-arrestin2 KO mice expressing CD11b.** Upper panels: populations of cardiac myeloid cells are shown. Gated area was defined as viable cells. FSC: Forward scatter, SSC: Side scatter. Lower panels: Expression of CD11b in viable cardiac macrophages. Percentages of CD11b-positive cells are shown. Control represents cardiac macrophages that are not treated with anti-CD11b antibody.
(TIF)
