# Supplementary figures and images for: β-arrestin2 in Infiltrated Macrophages Inhibits Excessive Inflammation after Myocardial Infarction
Source: PLoS One. 2013 Jul 8;8(7):e68351. doi: 10.1371/journal.pone.0068351 (PMC3704591; doi:10.1371/journal.pone.0068351)

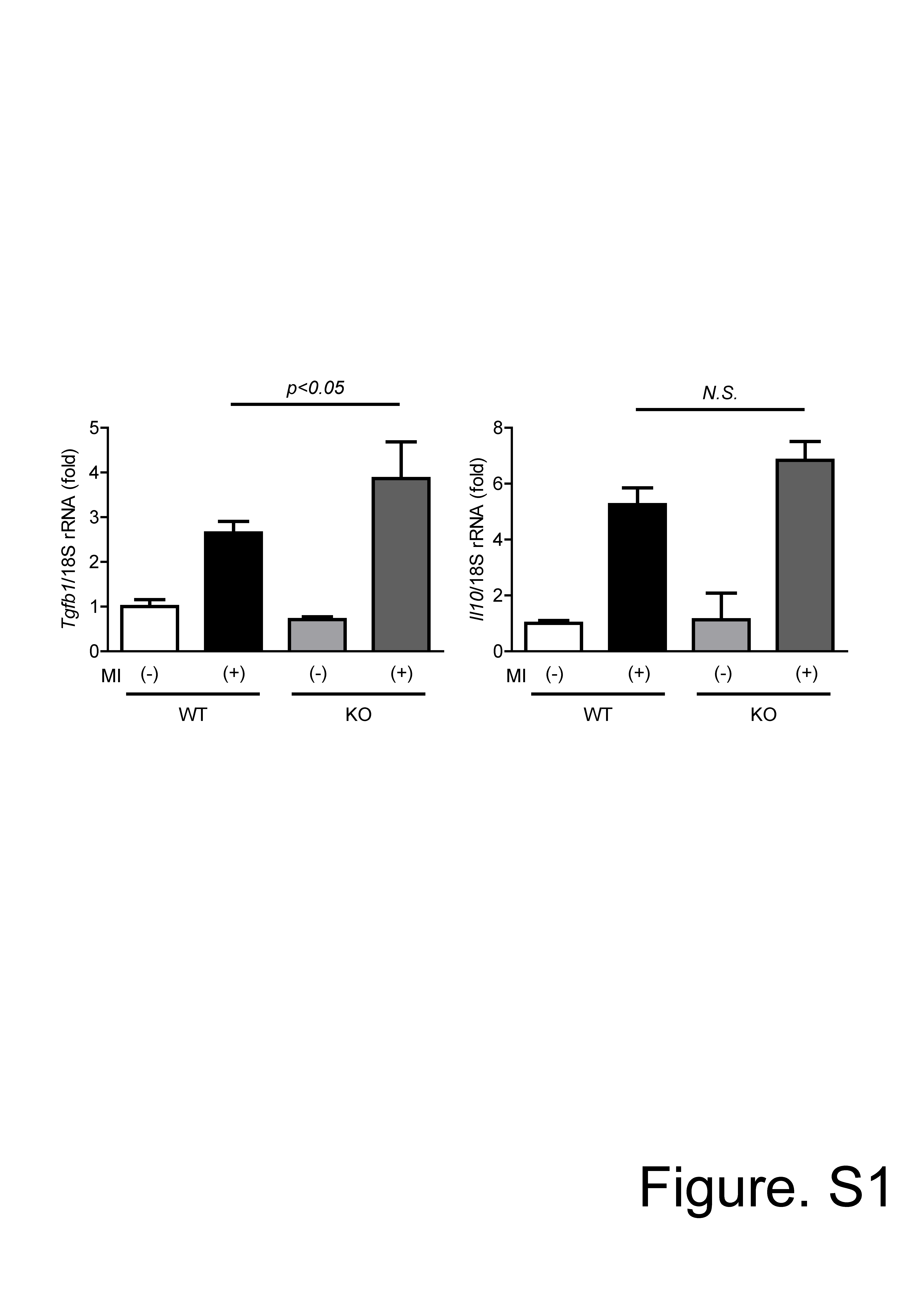

Supplement: Figure S1 — mRNA expression levels of anti-inflammatory genes, TGF-β1 ( Tgfb1 ) and IL-10 ( Il10 ), in the hearts of WT and β-arrestin2 KO mice at 3 days after MI. Total RNA extracted from sham-operated ventricles or MI-operated infarct area were subjected to real time RT-PCR. (TIF) [file pone.0068351.s001.tif]

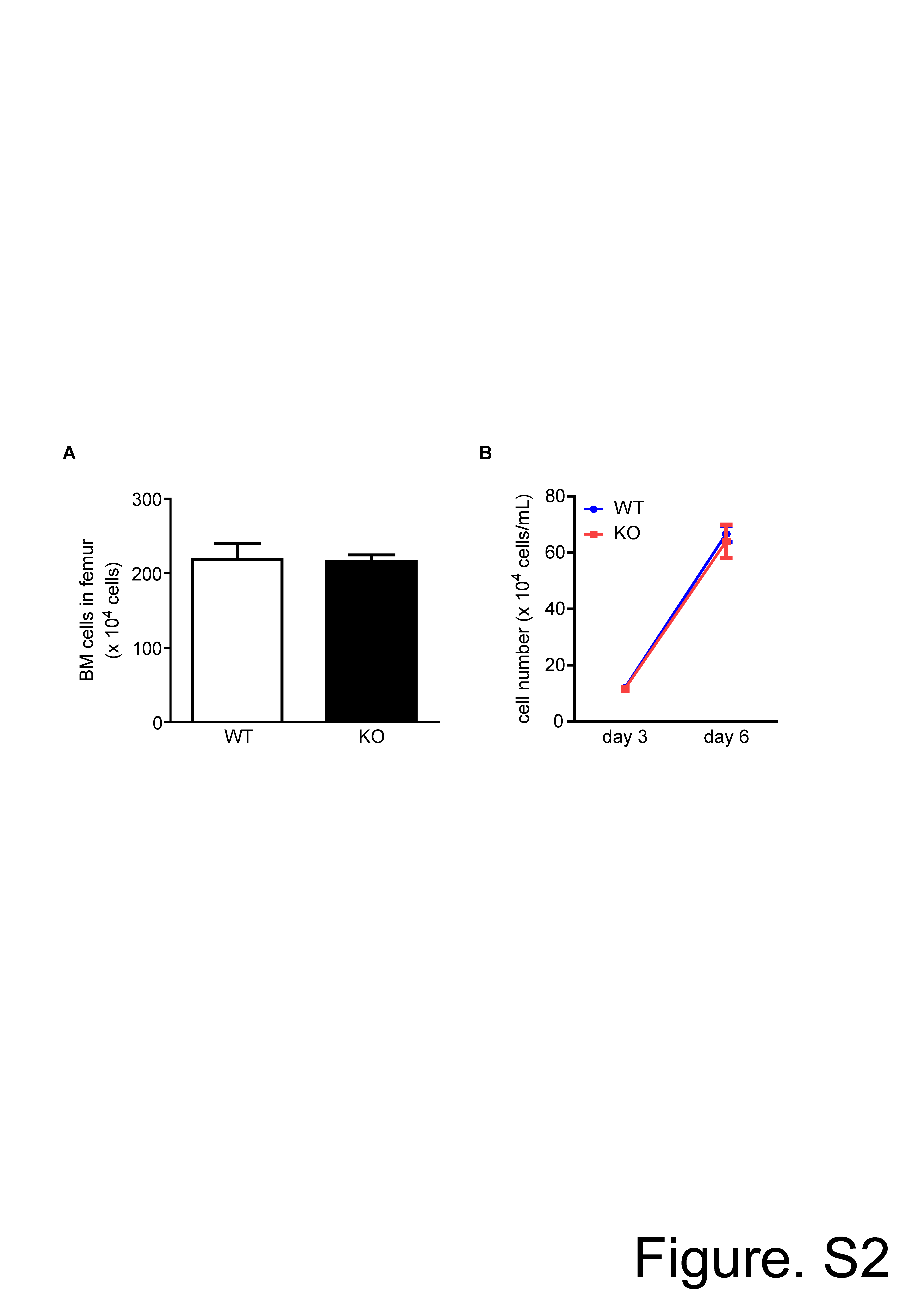

Supplement: Figure S2 — Numbers of bone marrow (BM) cells collected from a femur of WT and β-arrestin2 KO mice ( A ). n = 3 per each group. (B) Differentiation of BM-derived cells from WT and β-arrestin2 KO mice into BM-derived macrophages. BM-derived cells plated on the non-treated dishes were cultured in the α-MEM containing 10% FBS, and macrophage colony-stimulating factor on 6 cm plate. They were detached and the number of BM-derived cells was counted at 3 or 6 days after plating. n = 4 per each group. (TIF) [file pone.0068351.s002.tif]

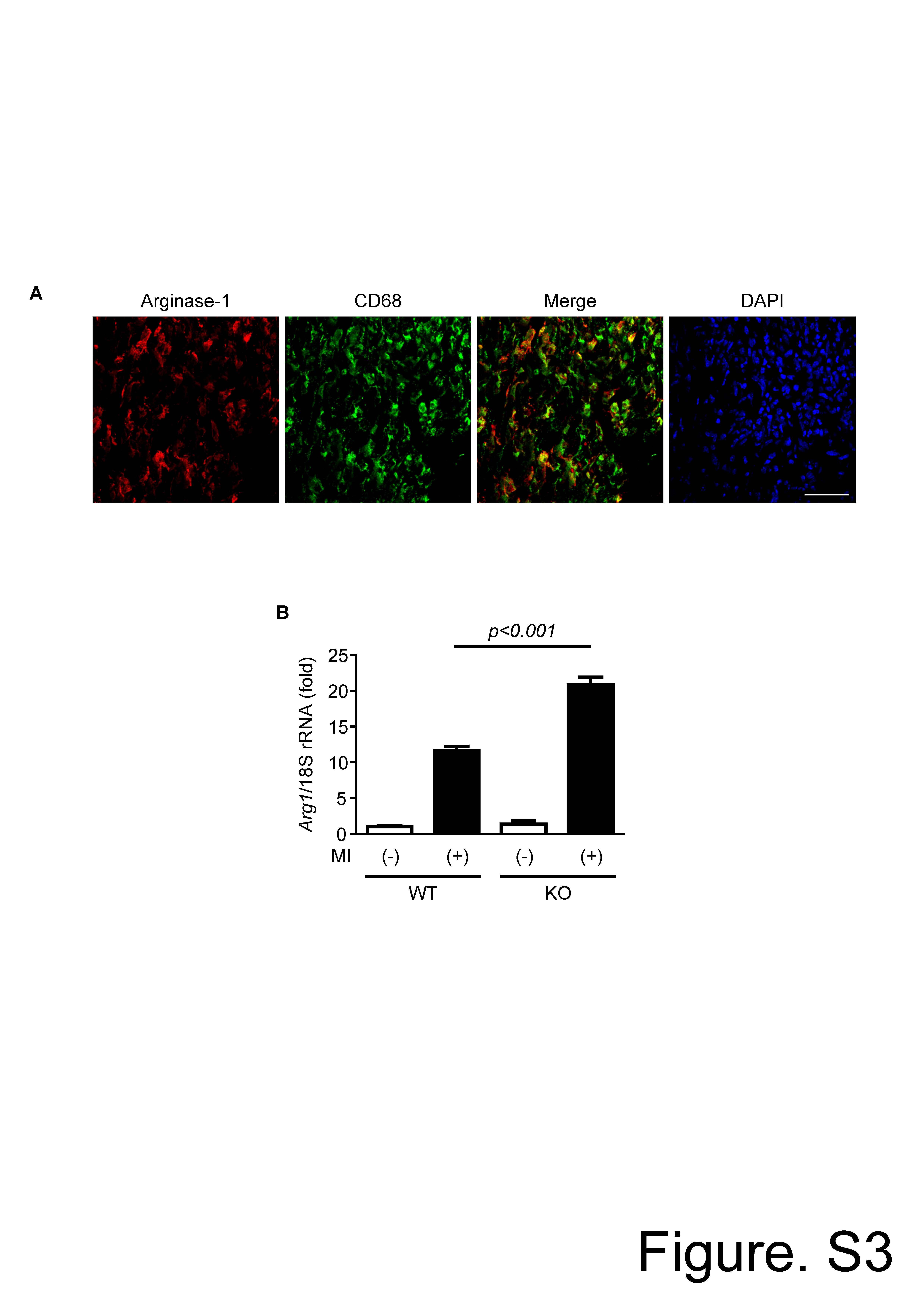

Supplement: Figure S3 — Immunohistochemical analysis of the expression of Arginase-1 on the heart section of MI-operated WT mice ( A ). CD68 and DAPI were used as markers of monocytes/macrophages and cell nuclei, respectively. Scale bar, 50 µm. (B) mRNA expression level of Arginase-1 (Arg1) 3 days after MI in the heart of WT and β-arrestin2 KO mice. (TIF) [file pone.0068351.s003.tif]

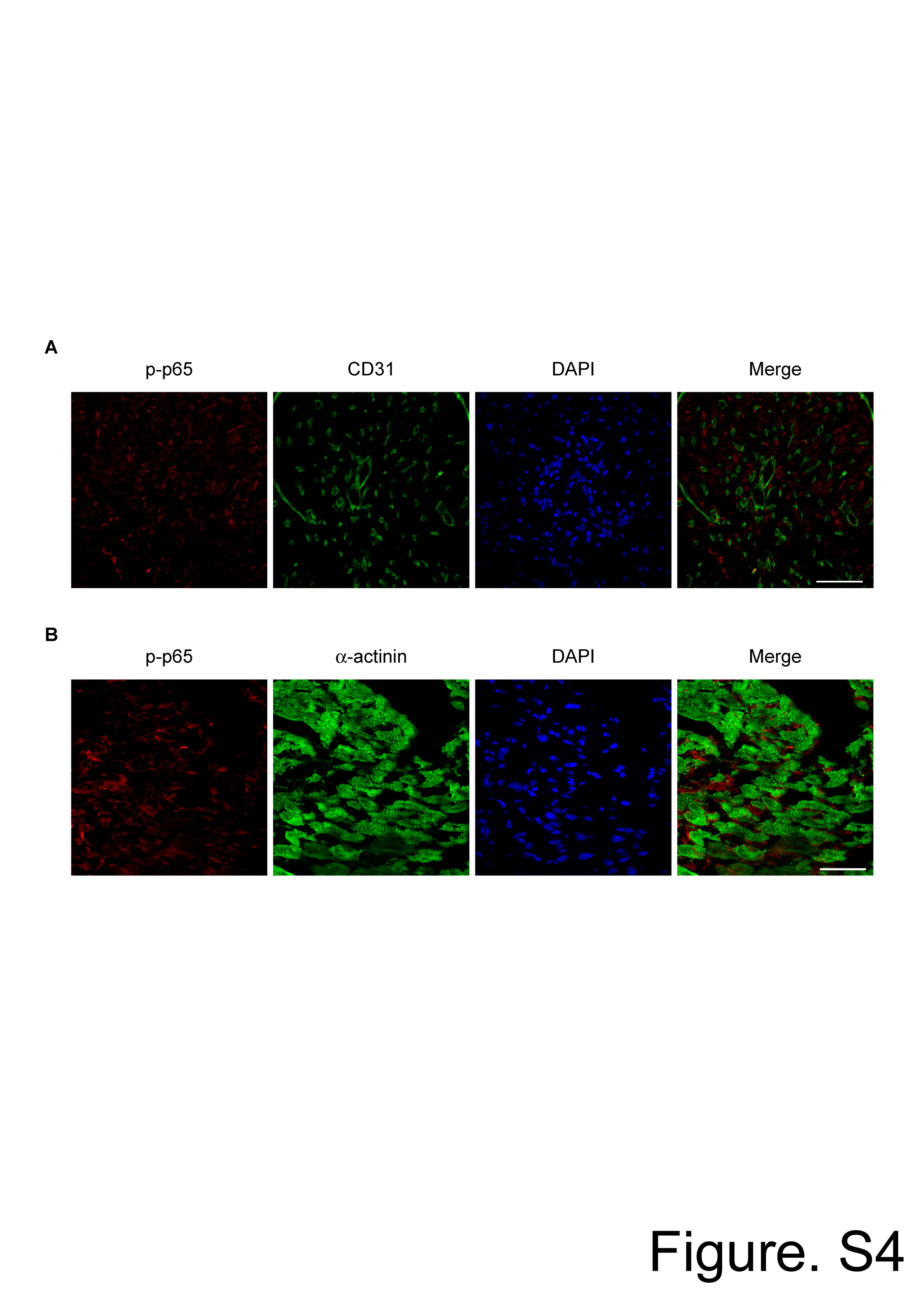

Supplement: Figure S4 — Immunohistochemical analysis of p65 phosphorylation in endothelial cells and cardiomyocytes. Phospho-p65 (p-p65) (red) was co-stained with (A) CD31 or (B) α-actinin (green) at the infarct area on the heart section of WT mice at 3 days after MI. Scale bar, 50 µm. (TIF) [file pone.0068351.s004.tif]

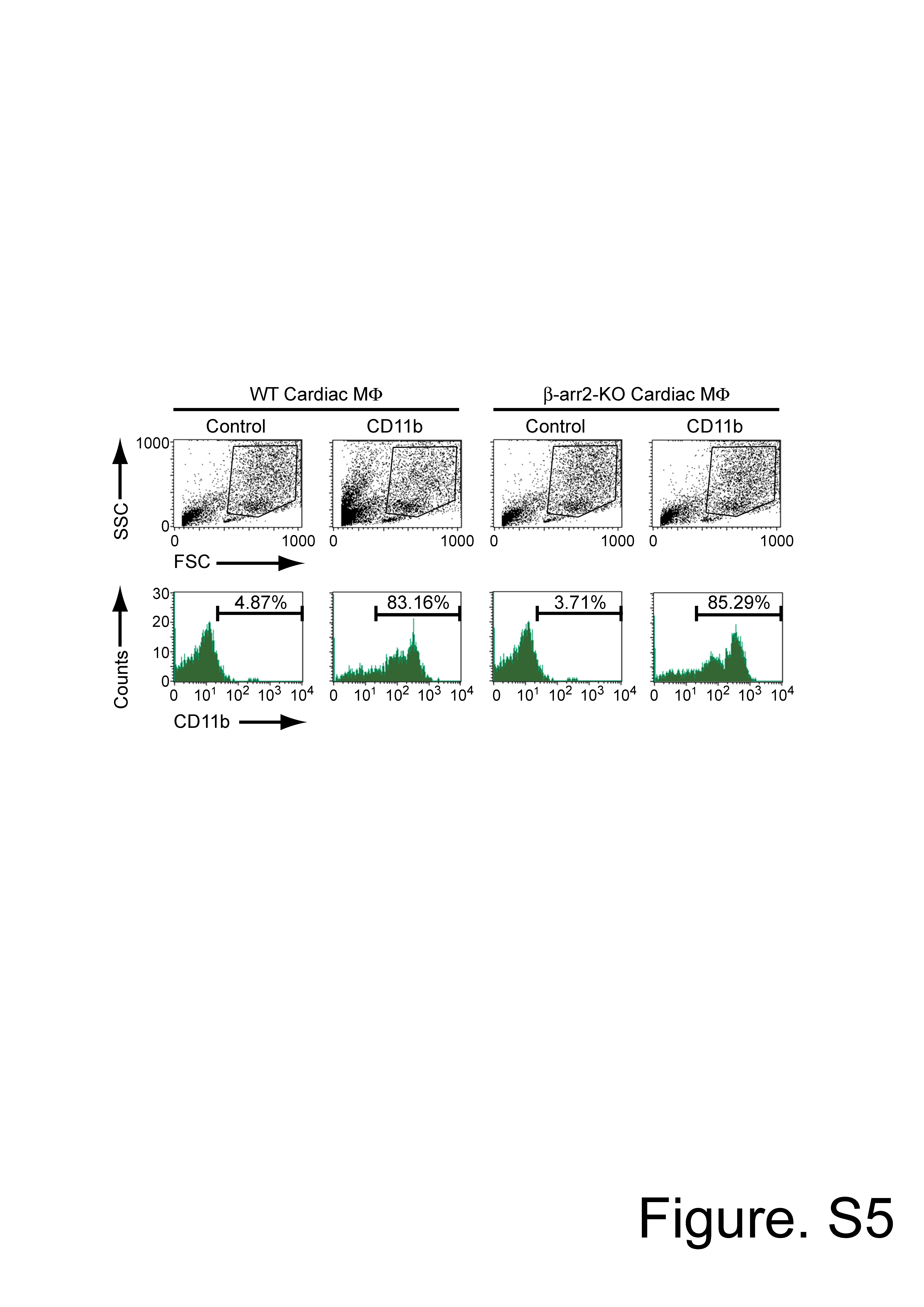

Supplement: Figure S5 — Flow cytometry of cardiac macrophages from WT and β-arrestin2 KO mice expressing CD11b. Upper panels: populations of cardiac myeloid cells are shown. Gated area was defined as viable cells. FSC: Forward scatter, SSC: Side scatter. Lower panels: Expression of CD11b in viable cardiac macrophages. Percentages of CD11b-positive cells are shown. Control represents cardiac macrophages that are not treated with anti-CD11b antibody. (TIF) [file pone.0068351.s005.tif]
